# Clinical Convictions Respecting Ascites, Expressed in the Form of Propositions

**Published:** 1850-08

**Authors:** 


*Clinical Convictions respecting Ascites, expressed in the form of Propositions.* By Dr. Dubini.—1. When ascites is not the result of a preexisting, or present, acute or chronic primary peritonitis, it will be found to depend upon hypertrophy with degeneration of the hypochondriacal viscera, the presence of abdominal tumours, a prior or existing dysentery, or upon *cirrhosis* of the liver.

2. In fact, peritonitis, depending on these various conditions of the viscera, is almost always the efficient cause of the effusion. In respect to the operation of visceral hypertrophy and abdominal tumours in inducing it, there can be no doubt. In dysentery, with ulceration of the mucous membrane, inflammation of the peritoneal coat is found. In *cirrhosis*, observation would seem to show, that the fluid may collect in consequence of peritonitis, or, on the other hand, mechanically from obstruction of the capillaries of the liver.

3. Post-mortem examinations prove, that defective absorption by the lymphatics, as a cause of dropsy, is a scholastic fable. Atony may follow the distension and destruction of tissues in the dropsical, but it is never, or with exceeding rarity, primary.

4. Ascites must not be confounded with other serous effusions, especially with general anasarca. The common causes of general dropsy, as hydræmia, scarlatina, Bright's disease, suppressed perspiration, &c., are not the direct causes of ascites, which, as already observed, has its own special cause, and seems also to recognise other directly or indirectly predisposings ones, such as intermittent fevers, abuse of spirituous drinks, and a certain inelastic and relaxed state of the organic tissues.

5. Diseases of the heart, so frequent a cause of anasarca, are never the primary cause of ascites, and if the two are found in coexistence, it is a mere coincidence. A diseased heart may lead to a hypertrophied or degenerated state of the liver, and this may give rise to ascites.

6. In enormous and old ascites, in which long and forcible pressure has been exerted upon the viscera by the fluid, a shortening of the intestinal tube takes place. In one case, the entire length of the canal was not more than three times that of the body.

7. The treatment by means of drastics is prompt, and always much desired by the patient, who wishes to relieve the distension as rapidly as possible. It is rare for them, however, to produce permanent cure, and the relief they at first afford is often followed by a relapse, under the influence of which the patient quickly finds himself in a worse state than before. If a red, shining, or excoriated tongue indicate a preexisting diarrhea, in place of a relapse, drastics will cause irreparable mischief, by provoking a second and artificial diarrhea, which will only cease with life itself.

8. Diaphoretics, employed in this generally non-febrile disease, are praised in the books: but what practitioner ever prescribed them for apyretic ascites? Nature alone, in particular cases, contrary to our physiological laws, induces simultaneous profluvia of sweat and urine, until the ascites is totally dispersed. Iodine and mercurial frictions are but therapeutical delusions, although in certain cases, in which the symptoms of peritonitis still exist, calomel may be of service. The frequent failure of diuretics is well known.

9. When even the local abstraction of blood is no longer admissible, and it is hence presumable that the peritonitis no longer exists, *compression* of the abdomen by a bandage is a useful aid to diurectics. Like all other means it may be misused, but twelve years' experience of it leads the author to state, that if the dropsy be not encysted, and the lower extremities have not become edematus, compression will not only in the majority of cases remedy the effect, but frequently also remove the morbid cause of the effusion. In very many cases, he has seen the same diuretics, which had long been uselessly employed, succeed admirably as soon as a methodical compression was conjoined to their use.

10. After recovery, when the ascites has depended upon dysentery or primary chronic peritonitis, a relapse may be prevented by opening an issue, at the inner side of the leg, just below the knee. After the application of a very small blister, a minute ivory ball may be compressed against the denuded surface to effect this.

11. There are certain cases of anasarca, from *hydræmia*, in which the ascites though not primary is very considerable, and threatens suffocation from its rapid increase. In these there is always found a degree of hypertrophy of the liver, a section exhibiting it of a bright yellow color, and of the consistence of soft chalk. The blood is scarcely red, and very watery. All the symptoms of chlorosis, and some of those of scorbutic are present, as palpitation, syncope, vertigo, tinnitus, muscular debility, pallor of the mucous membranes, anorexia, thirst, constipation, paucity of urine, and sweating, with frequent attacks of epistaxis. In these cases, the *scuffle* heard after the first sound of the heart is rather referable to the serous crasis of the circulating fluid, than to any defect in the circulatory organ. For this train of symptoms the author had long sought medicinal substances, which, while they proved unstimulating diuretics, might remedy the condition of the blood without disposing the organism too much to a state of phlogosis, which, in subjects of hydræmia, always terminates in new effusions, that in their result prove fatal, if they do not so in their immediate consequences. What he terms a "martial lemonade," has best fulfilled by its effects the chief indications, and especially by causing a free diuresis. It is formed by dissolving six grains of *sulphate of iron* in a pound and a half of water sweetened with an ounce of syrup, and adding a drachm of *sulphuric acid*—the period and continuance of its administration, and the dose in which it is given, being regulated by the practitioner, according to the exigencies of the case. Its use is contra-indicated in those cases in which the tongue is red and glazed, or has prominent papille, and when a certain amount of febrile action indicates the presence of thoracic inflammation or of hepatic peritonitis.—*Bulletino delle Scienze Mediche*, ser. 3, tom. xv, pp. 343–51.

